# Estuarine Aquacultures at the Crossroads of Animal Production and Antibacterial Resistance: A Metagenomic Approach to the Resistome

**DOI:** 10.3390/biology11111681

**Published:** 2022-11-21

**Authors:** Daniel G. Silva, Célia P. F. Domingues, João F. Figueiredo, Francisco Dionisio, Ana Botelho, Teresa Nogueira

**Affiliations:** 1 INIAV—National Institute for Agrarian and Veterinary Research, IP, 2780-157 Oeiras, Portugal; 2 cE3c—Center for Ecology, Evolution and Environmental Changes & CHANGE—Global Change and Sustainability Institute, 1749-016 Lisbon, Portugal

**Keywords:** resistome, metagenomics, aquaculture farms, mobile genetic elements, resistance fingerprint

## Abstract

**Simple Summary:**

The overuse of antibiotics in human and animal health has been favoring antibiotic resistance in bacteria. Moreover, antibiotic-resistance genes can spread in microbial communities, between bacteria, either pathogenic or commensal, by mobile genetic elements. The rise of aquaculture farms, to overcome the growing demand for fresh fish, can lead to the overuse of antibiotics to control diseases and promote growth. This work presents a first snapshot of the antibiotic resistance genes that are present in the sediments of oyster-extensive and gilthead bream semi-intensive aquacultures located in estuaries of three important rivers in the north, center, and south of Portugal. The metagenomic analysis approach revealed that the most diverse categories of antibiotic resistance are macrolide, tetracycline, and oxazolidinone classes. These resistances can hamper the effective treatment of infections in humans if transmitted through the food chain.

**Abstract:**

It is recognized that the spread of antibiotic resistance (AR) genes among aquatic environments, including aquaculture and the human environment, can have detrimental effects on human and animal health and the ecosystem. Thus, when transmitted to the human microbiome or pathogens, resistance genes risk human health by compromising the eventual treatment of infections with antibiotic therapy. This study aimed to define the resistance profile of aquaculture farms and their potential risk for spreading. Twenty-four sediments from oyster and gilthead sea bream aquaculture farms located in three Portuguese river estuaries (17 sediments from Sado, 4 from Aveiro, and 3 from Lima) were studied by comparative metagenomic analysis. The computation of the diversity of genes conferring resistance per antibiotic class revealed a significant increase in aminoglycosides, beta-lactams, disinfectants, quinolones, and tetracyclines counts. In all geographic locations under study, the most diverse AR genes confer resistance to the macrolides, tetracyclines and oxazolidinones classes, all of which are medically important for human and animal therapies, as well as resistance to disinfectants. The diversity of mobile genetic elements correlated with the number of AR genes such as tetracyclines, suggesting that AR could be easily mobilized among bacterial genomes and microbiomes.

## 1. Introduction

The spread of antibiotic-resistance (AR) genes in the environment can be promoted by anthropogenic activities, such as land animal husbandry and aquaculture [1]. The leading aquaculture producer in Europe is Norway, the eighth main producer worldwide. Portugal, as a traditional fishing country, is the highest consumer of sea products in the European Union (EU) (55.6 kg/per capita/year versus 22.3 kg/per capita/year in the EU), and the third in the world, after Iceland and Japan [2]. Since it is impossible to increase the fishing quota to avoid the exhaustion of natural resources, the alternative is to improve aquaculture production. Portugal has invested in the development of aquaculture for the last decade, attaining, in 2018, 13,000 tons produced, making it the 16th main producer in the EU that year [3]. Most Portuguese aquaculture facilities focus on the production of marine fish and bivalve mollusks and operate essentially in estuaries and coastal lagoons, in extensive or semi-intensive systems. This is the case for the extensive production of clams and oysters that corresponded to over three-quarters of total mollusk production in Portugal in 2018 [4].

This rapid growth in aquaculture will probably be accompanied by a rapid increase in therapeutic and prophylactic usage of antibiotics, as maturation in captivity promotes the onset of bacterial infections [5], including those important in human therapeutics, which raises concerns regarding the potential risks to public health. These drugs are usually present, in the water and the sediments, at subtherapeutic levels for prolonged periods leading to selective pressure, suspected to promote the occurrence and spread of AR genes in aquaculture and related environments.

Furthermore, spillover between and within marine and terrestrial food production sectors occurred, along with the flow of microorganisms, antibiotics, antibiotic residues, and AR genes [6], and the same resistance patterns have been seen in land animal husbandry and aquaculture [7]. Therefore, antimicrobial resistance can develop in aquatic bacteria due to antibiotic therapy or to contamination of the aquatic environment with human or animal waste [8]. Antibiotic and antibiotic-resistant bacteria in aquaculture systems are likely to last for a long time and be transferred to humans through the contamination of products for human consumption [9]. Urban rivers have already been described as hotspots of antibiotic resistance [9], and, according to Xu and colleagues, river sediments harbor higher levels of antibiotic-resistance genes than aquaculture ponds [10,11]. 

High-throughput genomic technologies offer new approaches namely for monitoring environmental, animal, and human health. Because these techniques are culture-independent, they can access genomic information from most bacteria that are not culturable, corresponding to 99% of all the bacteria in a sample. Shotgun whole-genome sequencing metagenomics has also been used to investigate bacterial resistance and to capture a broad diversity of resistance genes [12]. In this way, it is possible to describe the abundance and diversity of resistance genes in the sediments of the oyster and gilthead bream farms and better understand the dynamics of antimicrobial resistance in these and similar environments. 

Furthermore, another objective of this study was to evaluate the potential of transference between bacteria of the identified resistance genes. There is currently limited information on the occurrence and genomic mobilization of antimicrobial resistance in aquaculture environments, not only in Portugal but also in the world, with the exception of China [13,14,15]. A recent meta-analysis study of 460 published articles revealed that the aquaculture sector is a reservoir of resistance to antibiotics with therapeutic potential [16].

Several studies have suggested that stressors such as antibiotics could contribute to a general mobilization of genetic material in bacterial communities, and, therefore, genes involved in genetic mobility such as plasmids [17,18] were specifically looked for. It must be noticed that, with this metagenomic approach, it is impossible to directly link mobile elements, such as plasmids or integrons, to specific bacterial resistance genes. However, the amount and diversity of the mobile genetic elements in metagenomes may correlate with the potential risk of the mobilization of antimicrobial resistance genes within the aquaculture and into the environment. 

Portuguese oyster and gilthead sea bream aquacultures in the Sado River estuary and oyster aquacultures in the Lima River estuary and Aveiro Lagoon were selected for this study due to their importance and volume of production. Oyster aquacultures have been growing in the last few years in Portugal, and only a few studies have been performed to mitigate their impact on the ecosystem [19]. A recent study has focused on the selection of antibiotic resistance by metals in riverine bacterial communities in Portugal [20]. However, there is still a need to understand the dynamics of antibiotic-resistance genes and their transmission potential in these environments. 

For this purpose, this study aims to evaluate the profile of resistance genes in estuarine aquaculture environments, located in three different regions of Portugal, and identify those that may pose hazards to human health, either by their relative abundance and genetic diversity or by their potential to spread.

## 2. Methods

### 2.1. Sample Collection and Preparation

**Figure 1:**
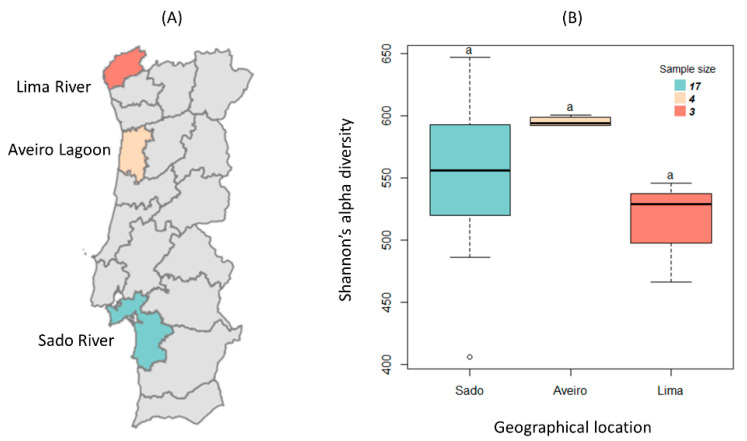
Distribution of Shannon’s alpha diversity of each metagenome by geographic location of the river or estuary from which they originate: Sado, Aveiro, and Lima. (**A**) Geographical regions under study shown on a map of Portugal. (**B**) Each boxplot represents a geographical location, namely the Sado River (blue), Aveiro Lagoon (beige), and Lima River (salmon). The sample sizes are 17, 4, and 3, respectively. The bottom and top of the boxplot represent the first and third quartiles, the horizontal line is the median, and the vertical dashed lines are the 1.5 interquartile range. The black circles represent the outliers. Kruskal–Wallis’s analysis indicated that there are no significant differences in Shannon’s alpha diversity between the three geographical locations, as indicated by the letter a (*p*-value > 0.05).

Samples of oyster aquaculture sediments were collected between November 2018 and July 2019 in three estuarine regions: Lima River near Viana do Castelo city in the north, Aveiro Lagoon near Aveiro city in the center, and Sado River near Setúbal city in the south of Portugal. Sediments of gilthead sea bream were also collected in the Sado Estuary. The geographical distribution of the sampling sites is shown in Figure 1A.

All procedures related to the AquaRAM project samples are described in Supplementary File S1 [21,22,23,24]. 

### 2.2. Genomic Analysis

Twenty-four Illumina raw sequence data file pairs in fastq format were assembled on the MG-RAST metagenomic analysis server [24] (project mgp95904-https://www.mg-rast.org/mgmain.html?mgpage=search&search=mgp95904, accessed on 16 November 2022). The pipeline options chosen were the removal of artificial replicate sequences, any host-specific Homo sapiens NCBI v36 specie sequence, and low-quality sequences. The lowest Phred scores to count as a high-quality base was set to 15 and trimmed to at most 5 low Phred score bases. FASTA formatted files (299.screen.passed.fna) that passed DNA contamination removal, and those containing the derived protein-coding sequences clustered at 90% identity (550.cluster.aa90.faa), were retrieved programmatically from the MG-RAST file formatting pipeline-interface (API) in a Linux environment. ResFinder and Mustard databases of antibiotic-resistance genes were used in this study. ResFinder is a comprehensive and up-to-date database of acquired genes and chromosomal mutations mediating antibiotic resistance in total or partial DNA sequences of bacteria of major public health relevance. This database is divided into 17 categories and was downloaded from https://bitbucket.org/genomicepidemiology/resfinder_db.git, accessed on 30 October 2020 [25]. Mustard is a database of antibiotic resistance determinants that was generated using a 3-dimensional modeling-based approach, called pairwise comparative modeling (PCM), which accurately predicts functions of proteins that are distantly related to proteins with known functions from the human intestinal microbiota. The Mustard database was downloaded from http://mgps.eu/Mustard/db/all_ard.zip (accessed on 26 October 2020) [26]. Files containing sequences of integrase, relaxase, and transposase proteins were generated on 26 November 2021 from the Swiss-Prot database (https://www.uniprot.org/, accessed on 16 November 2022) using the taxonomy filter for bacteria.

The identification of all genes and gene product orthologues against the abovementioned databases was performed by NCBI-blast-2.10.1+-1.x86_64 (National Library of Medicine, Bethesda, MD, USA) (downloaded on 8 June 2020) blast alignments in a Linux environment. For the alignment of DNA sequences, E values < 10^−5^, 60% query coverage, and 90% identity as a filter were used. For the alignment of protein sequences, filter values E < 10^−5^, 60% of query coverage, and 30% of identity were applied.

The number of orthologues of each of the abovementioned gene/protein categories was computed. Finally, we computed the “size” of each metagenome, as the number of nucleotides in the DNA fasta files, and the number of proteins in the clustered protein fasta files. The values obtained were used to normalize the number of orthologues per metagenome. 

PlasmidFinder is a tool that allows the identification of plasmids from bacterial species of the *Enterobacteriaceae* family and Gram-positive bacteria. This tool was used to find, analyze, and annotate the plasmids present in the samples under study, with a minimum identity and coverage of 10% [27]. The PlasmidFinder database was downloaded from https://bitbucket.org/genomicepidemiology/plasmidfinder_db.git, accessed on 2 December 2021.

### 2.3. Statistical Analysis

Kruskal–Wallis’s test was used to perform the variance analysis on the effect of the geographic location of the samples, followed by a Dunn post hoc analysis. Kruskal–Wallis’s test belongs to the stats package, version 4.1.2. The Wilcoxon rank-sum test was used to test whether there are differences between higher and lower relative frequencies of orthologous antibiotic resistance gene counts. Both Kruskal–Wallis’s and Wilcoxon’s test belongs to the *stats* package, version 4.1.2; *p*-values less than 0.05 were considered statistically significant. Both tests were performed with R—version 3.5.1 [28] and used the FSA package [29] to perform Dunn’s test [30].

## 3. Results

### 3.1. Sample Characterization

The three estuarine regions chosen are located near Portuguese cities and therefore have anthropogenic contact (Figure 1A) [21]. After collecting samples of aquaculture sediments (underneath the nets and bags where oysters and gilthead bream are cultivated and where antibiotics and their active residues are deposited), DNA was extracted and shotgun Illumina whole-genome sequencing was performed, and the paired-end fastq files were analyzed in the MG-RAST pipeline, which can be assessed at https://www.mg-rast.org/mgmain.html?mgpage=search&search=mgp95904 (accessed on 16 November 2022) and [21]. There were 4 samples collected from an oyster farm in the Ria de Aveiro, 3 collected from an oyster farm in the Lima River, and 17 collected from the Sado River, of which 4 are from a sea gilthead bream and 13 are from an oyster farm.

A comparison of the alpha diversity [21] distribution of the multiple microbiomes between the different geographic locations was performed and it was concluded that there are no significant differences (Figure 1B). This result, however, does not allow us to conclude that the metagenomes in the different locations have a similar phylogenetic composition, but only that they have a similar number of different bacterial clades. It allows, nonetheless, for assuming that the increase in diversity of AR determinants and MGEs is not influenced by the microbial diversity of each metagenome.

During this study, two types of files were used: fasta files of assembled and annotated DNA contigs sequences and derived files containing protein sequences clustered at 90% identity. Therefore, searching for orthologues in each of these two types of files generates different information: searching for orthologues using DNA measures the quantity/abundance of a given determinant (hereafter identified as “Gene”), whereas searching for orthologues of “proteins”, as they are clustered, allows diversity to be measured (hereafter identified as “Protein Diversity”).

### 3.2. Antibiotic Resistance Encoding Genes

**Figure 2:**
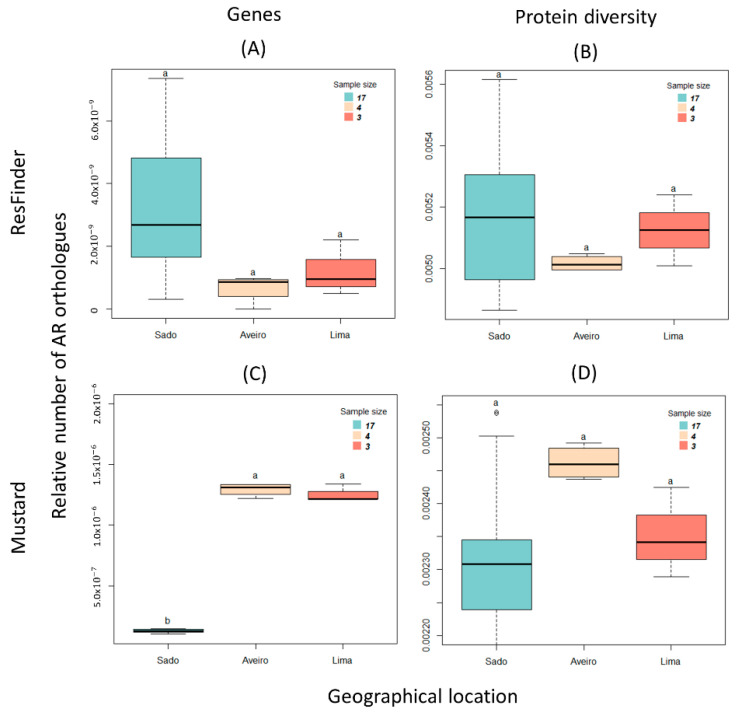
Distribution of antibiotic resistance orthologues by the geographical location of the river or estuary from which they originate: Sado, Aveiro, and Lima. (**A**) A relative number of antibiotic resistance genes using the ResFinder database; (**B**) diversity of AR proteins using the ResFinder database; (**C**) a relative number of antibiotic encoding resistance genes using the Mustard database; and (**D**) diversity of AR proteins using the Mustard database. Each boxplot represents a geographical location, namely the Sado River (blue), Aveiro Lagoon (beige), and Lima River (salmon). The sample sizes are 17, 4, and 3, respectively. The bottom and top of the boxplot represent the first and third quartiles, the horizontal line is the median, and the vertical dashed lines are the 1.5 interquartile range. The black circles represent the outliers. Kruskal–Wallis’s analysis followed by post hoc comparisons using Dunn’s test (see main text) indicated that there are no significant differences between the relative number of determinants of AR between the different geographical locations for (**A**), (**B**)**,** or (**D**). In (**C**), there is a significant difference between the Sado River and the Aveiro Lagoon, and the Lima River, as indicated by the two different letters a and b (*p*-value < 0.05).

To identify, classify, and quantify AR encoding genes (hereafter referred to as AR genes), the Basic Local Alignment Search Tool (BLAST) software was used to align DNA and protein sequences from each of the metagenomes under analysis against two AR databases: ResFinder, which consists of 3160 acquired AR genes organized into 17 categories, including disinfectant resistance, and Mustard, which is a catalogue of 3.9 million proteins from the human gut microbiome. Larger metagenomes can be expected to contain more AR orthologues. To remove this effect, the counts were divided by the total number of nucleotides in the case of DNA sequences, and by the total number of proteins in the case of protein sequence files. Figure 2 shows the boxplots of the relative number of AR determinants grouped by the geographical location of the river estuary or lagoon from which they originate: Sado, Aveiro, and Lima. 

The relative number (Figure 2A) and diversity orthologues (Figure 2B) of AR in the ResFinder database were computed. Kruskal–Wallis’s test shows that there are no statistically significant differences in the ”Protein diversity” at the three sites (*p*-value = 0.543 for “Protein Diversity”), but statistically significant difference for the number of “Genes” (*p*-value = 0.034) (Table 1). The Dunn test for multiple comparisons, with *p*-values adjusted with Holm’s method, shows a marginally significant difference (*p*-value = 0.06) between the orthologous gene counts of Aveiro Lagoon and of Sado River.

Regarding the distribution of the relative number (Figure 2C) and diversity (Figure 2D) of orthologues in the Mustard database, Kruskal–Wallis’s test reveals that the number of orthologous genes between the three geographical locations is significantly different (*p*-value = 0.001) (Table 1). The Dunn test for multiple comparisons, with *p*-values adjusted by using Holm’s method, shows that the differences between Aveiro Lagoon and Sado River orthologous gene counts are statistically significant (*p*-value = 0.004) (Table 1). This result suggests that the microbiome of aquaculture sediments belonging to the Aveiro Lagoon and the Lima River are enriched with microorganisms belonging to the human gut microbiome compared to those from the Sado River. On the other hand, in the microbiome from the Aveiro Lagoon, the diversity of proteins conferring resistance to antibiotics is slightly higher, with results marginally supported by the Kruskal–Wallis’s test (*p* = 0.087), and the Dunn test between the Aveiro Lagoon and the Sado River (*p*-value = 0.082) (Table 1). 

**Figure 3:**
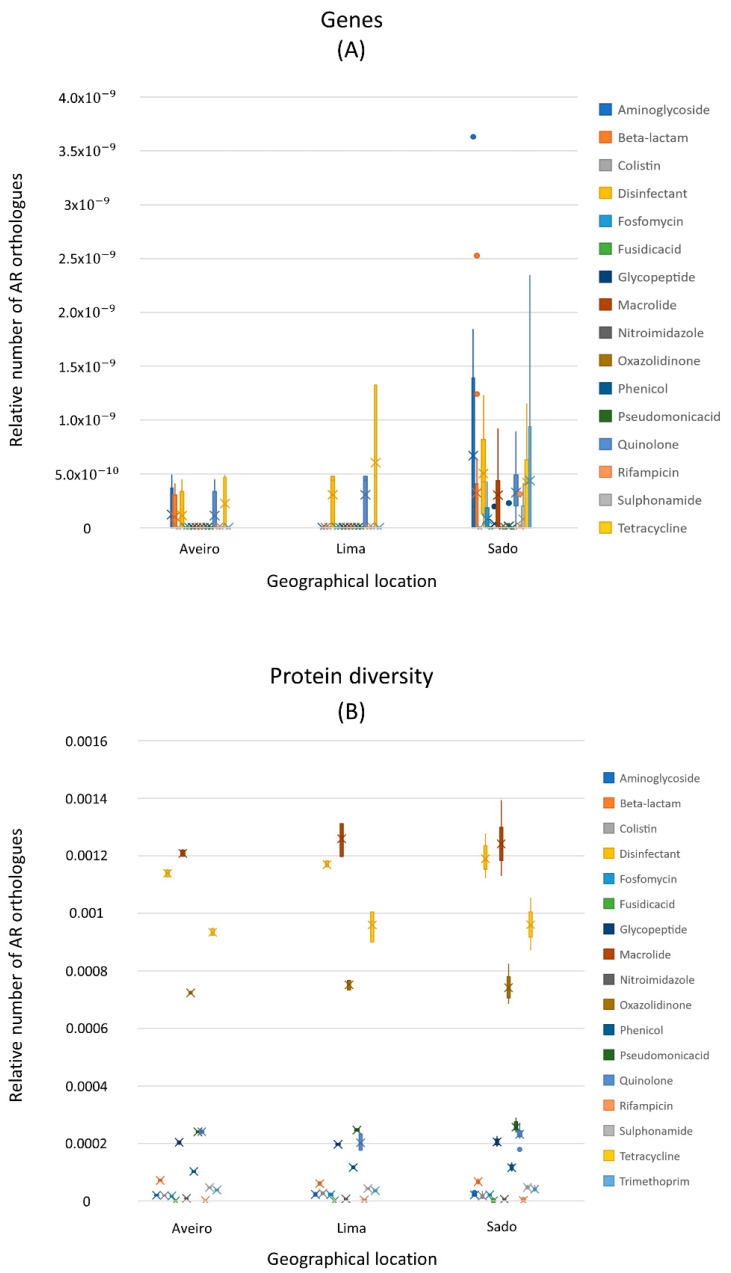
Frequency of different AR groups by antibiotic class according to the ResFinder database and by the geographical location of the river or estuary from which they originate: Sado, Aveiro, and Lima: (**A**) relative number of antibiotic resistance-encoding genes; (**B**) relative number of antibiotic resistance protein diversity. Each box plot represents the distribution of values: the bottom and top of the box are the first and third quartiles, the crosses are the medians, and the vertical lines are the 1.5 interquartile range.

The results described above reveal the distribution of the determinants of AR in each of the aquatic environments under study. However, it does not provide information on the resistance profile for each site. To perform the profiling, a category was assigned corresponding to the classification of each of the AR gene products on the ResFinder database (Figure 3). 

Preliminary assessment of the orthologous AR gene counts (Figure 3A) revealed that they fall into two groups: one with higher relative frequencies, referred to as “high” (comprising resistance to aminoglycosides, beta-lactams, disinfectants, quinolones, and tetracycline), and another with lower frequencies (comprising the remaining 12 categories), referred to as “low” (Figure S1). The computation of the diversity of AR orthologues (Figure 3B) also clustered into two groups: one with higher relative frequencies, referred to as “high” (comprising resistance to macrolides, tetracyclines, and oxazolidinones), and another with lower frequencies, referred to as “low” (Figure S1). To test the pertinence of this categorization on the differences between the two groups, boxplots were generated with all “high” and “low” counts both for the “Genes” and the “Protein Diversity”, and the Wilcoxon test was performed. Highly significant differences were found (W = 444, *p*-value = 0.001 for the “Genes” data sets (Figure S1A); and W = 576, *p*-value = 6.2 × 10^−14^ for the “Protein Diversity” data sets (Figure S1B). These results indicate that there is higher diversity of AR orthologues belonging to the disinfectant, macrolide, tetracycline, and oxazolidinone classes than AR orthologues belonging to other classes, in all the geographic locations under study. 

### 3.3. Distribution of the Mobile Genetic Elements

**Figure 4:**
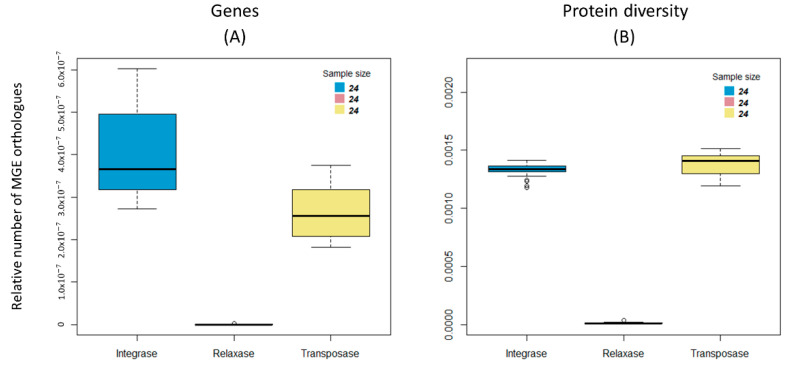
Distribution of the number of orthologues identified by category of mobile genetic elements (MGEs), normalized to metagenome size: (**A**) genes and (**B**) protein diversity. Each boxplot represents a category of MGE, namely integrase (blue), relaxase (pink), and transposase (yellow). All categories of MGE have a sample size of 24. The bottom and top of the boxplot represent the first and third quartiles, while the horizontal line represents the median, and the vertical dashed lines indicate the 1.5 interquartile range. The black circles represent the outliers.

Another very important dimension concerning the epidemic potential of bacterial genes in each metagenome is the evaluation of the ease with which bacteria in each microbiome share and spread genes among themselves, as well as between different microbiomes. With this idea in mind, the number of each MGE type was computed by identifying orthologues of relaxases to infer the number of mobile plasmids and conjugative elements, transposases as markers of transposons, and integrases as markers of integrons. Figure 4 shows the distribution of the different MGEs in this dataset.

Owing to the uneven sample size for the three separate locations, it was decided to focus on the 17 samples of the Sado River. It was not possible to identify many plasmids using the relaxases orthologue search as a proxy. Therefore, the PlasmidFinder software was also used to search for plasmids in the metagenomes under study. All the plasmids identified are listed in Table S1. The PlasmidFinder software was unable to identify many plasmids; therefore, the counts for this analysis were not used for the analysis. Among the six different plasmids that PlasmidFinder was able to find and identify and whose accession number is in column 2 (Table S1), is the multiresistance plasmid *pSCFS1* from *Staphylococcus sciuri* [31].

### 3.4. Spreading Potential of Antibiotic Resistance

**Figure 5:**
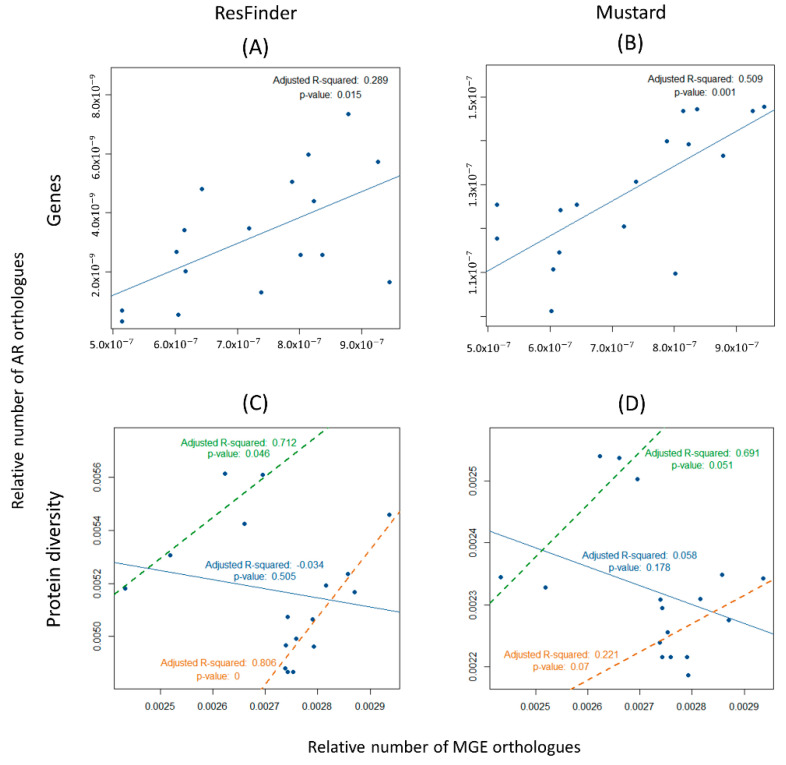
Correlation between antibiotic resistance (AR) orthologues and mobile genetic elements (MGEs) for the Sado River. Each point represents a metagenome. (**A**) Positive correlation between the relative number of antibiotic encoding resistance genes (ResFinder) and MGEs (Adjusted R-squared = 0.289, *p*-value = 0.015). (**B**) Positive correlation between the relative number of antibiotic encoding resistance genes (Mustard) and mobile genetic elements (Adjusted R-squared = 0.509, *p*-value = 0.001). (**C**) Negative correlation between the diversity of antibiotic encoding resistance proteins (ResFinder) and mobile genetic elements (Adjusted R-squared = −0.034, *p*-value = 0.505). The points agglomerate into two distinct clusters, leading to two positive correlations: one identified by the green regression line (Adjusted R-squared = 0.712, *p*-value = 0.046) and the other identified by the orange regression line (Adjusted R-squared = 0.806, *p*-value = 0). (**D**) Negative correlation between the diversity of antibiotic encoding resistance proteins (Mustard) and mobile genetic elements (Adjusted R-squared = 0.058, *p*-value = 0.178). The points agglomerate into two distinct clusters, leading to two positive correlations: one identified by the green regression line (Adjusted R-squared = 0.691, *p*-value = 0.051) and the other identified by the orange regression line (Adjusted R-squared = 0.221, *p*-value = 0.07).

The number of MGEs found, such as the mobilizable plasmids, conjugative elements, integrons, and transposons, are suggestive of gene mobilization between bacterial genomes in each environment (metagenomes). It was, therefore, investigated whether there is any correlation between ARs and MGEs that could evoke that resistance genes are genetically linked to these MGEs. If AR traits are frequently encoded on MGEs, the amount of MGEs would be expected to correlate with that of the number of AR genes across metagenomes. The counts performed using the DNA files provides an estimation of the number of AR genes and of the total number of MGEs identified by detecting orthologues of integrases, relaxases, and transposases, normalized by the size of each metagenome (number of nucleotides per metagenome). The scatterplots were generated, and linear regression model analyses were performed. Figure 5A,B show that there is a correlation between the amount of all MGEs and the amount of all ARs (Adjusted R-squared = 0.289, *p*-value = 0.015, for the ResFinder database; Adjusted R-squared = 0.509, *p*-value = 0.001, for the Mustard database). This correlation is stronger for the ARs that pertain to human gut microbiomes (Mustard database).

On the other hand, when the protein diversity data were analyzed (Figure 5C,D), this positive correlation between ARs and MGEs was no longer found (Adjusted R-squared = −0.034, *p*-value = 0.505, for the ResFinder database; Adjusted R-squared = 0.058, *p*-value = 0.178, for the Mustard database). These results show that the number of MGEs correlates with the number of genes (abundance), but not with the diversity of ARs. One may explain these results by postulating a specific association between some AR genes and some MGEs: these MGEs would move by replication across the microbiome, increasing the number of genes encoding these particular AR genes, but not their diversity (because the transferred genes are the same). 

**Figure 6:**
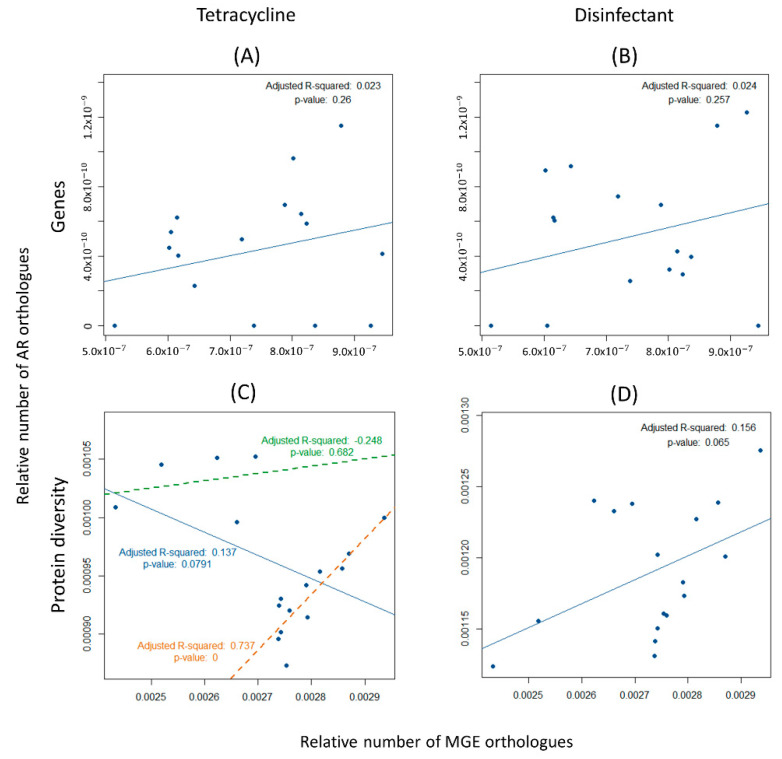
Correlation between antibiotic resistance (AR) orthologues for two ResFinder categories and mobile genetic elements (MGEs) for the Sado River. Each point represents a metagenome. (**A**) Positive correlation between the relative number of tetracycline encoding resistance genes and mobile genetic elements (Adjusted R-squared = 0.023, *p*-value = 0.26). (**B**) Positive correlation between the relative number of disinfectant encoding resistance genes and mobile genetic elements (Adjusted R-squared = 0.024, *p*-value = 0.257). (**C**) Negative correlation between the diversity of tetracycline resistance proteins and mobile genetic elements (Adjusted R-squared = 0.137, *p*-value = 0.079). The points agglomerate into two distinct clusters, leading to two positive correlations: one identified by the green regression line (Adjusted R-squared = −0.248, *p*-value = 0.682), and the other identified by the orange regression line (Adjusted R-squared = 0.737, *p*-value = 0). (**D**) Positive correlation between the diversity of disinfectant resistance proteins and mobile genetic elements (Adjusted R-squared = 0.156, *p*-value = 0.065).

As noted in the previous section, all the orthologues of AR genes from the ResFinder database were organized into their 17 categories. Additionally, the counts regarding the number of orthologues of tetracycline and disinfectant resistance genes were increased both as a relative amount and diversity (Figure 3). Therefore, the correlation between these genes and those of MGEs were studied separately. The results show that there is no correlation between the relative number of both tetracycline and disinfectant encoding resistance genes with MGEs (Adjusted R-squared = 0.023 and *p*-value = 0.26, for tetracycline resistance; and Adjusted R-squared = 0.024 and *p*-value = 0.257, for disinfectant resistance) (Figure 6A,B). Marginally significant results were found for the correlation between MGEs and the diversity, not only of the tetracycline-resistance proteins, but also of the disinfectant-resistance proteins and MGEs (Adjusted R-squared = 0.137 and *p*-value = 0.079, and Adjusted R-squared = 0.156 and *p*-value = 0.065, respectively) (Figure 6C,D). It is noteworthy that the correlation between MGEs resistance is negative in Figure 5C,D and Figure 6C. However, a close inspection of these figures suggests that the 17 points constitute two clusters, one with five points and another one with 12 points. This clustering is backed up biologically by the fact that the four gilthead bream samples belong to the five-point cluster, having just one oyster “outlier” sample. Therefore, the regression analysis of these data was performed, this time considering each of the two clusters separately. Positive correlations were obtained in all cases, even if some correlations are non-significant. For Figure 5C, a cluster of five points: Adjusted R-squared = 0.716 and *p*-value = 0.046; a cluster of twelve points: Adjusted R-squared = 0.806 and *p*-value = 0.00004; Figure 6C, cluster of five points: Adjusted R-squared = −0.248 and *p*-value = 0.682; a cluster of 12 points, Adjusted R-squared = 0.806 and *p*-value = 0.00004. For Figure 5D, a cluster of five points: Adjusted R-squared = 0.691 and *p*-value = 0.051; a cluster of 12 points, Adjusted R-squared = 0.221 and *p*-value = 0.070.

## 4. Discussion

### 4.1. Resistome Fingerprint of Sediments of Estuarine Aquacultures

The main aim of this study was to identify the AR profile in microbial communities from aquacultures from estuarine environments in Portugal. The reason aquacultures in natural environments but close to urban environments were chosen was to include the effect of environmental contamination on the genomic dynamics of microbiomes and their potential as reservoirs of AR genes. 

In the present study, sediment samples from three different geographical locations with similar microbial diversity were used. In all of them, the same group of AR genes accumulates, and their distribution differs from that of other resistance genes, a difference that is statistically significant (Figure 3 and Figure S1). It was thus possible to define the fingerprint of resistance in Portuguese aquacultures. If it were necessary to create a short list of genes, from aquaculture in estuarine environments in Portugal, worth monitoring for public health reasons, it would include those encoding resistance to macrolide, tetracycline, and oxazolidinone, together with resistance to disinfectants. These genes are those that share higher diversity in these samples, while the most abundant are those encoding for aminoglycoside, beta-lactam, disinfectant, quinolone, and tetracycline. The accumulation of these last genes in the metagenomes may result from clonal expansion. Previous studies show that sediments are biomes that possess a great diversity of antibiotic-resistance genes [32]. A comprehensive study of bacterial genomes reveals that, at the genomic level, the most frequent resistance genes present are those conferring resistance to beta-lactams, sulfonamide, quinolones, and only then to macrolides and tetracycline, and that resistance to oxazolidinone is rare [33]. This profile at the genomic level is different from that found in the metagenomes of aquaculture sediments, thus revealing a specific profile of these metagenomes.

Quinolones and tetracyclines (such as oxytetracycline) have been extensively used in aquaculture settings as prophylaxis, being administered mostly in food [8,34]. Furthermore, this group of genes, which are here called “high” (Figure 3 and Figure S1), confer resistance to antibiotics for animal and human use, revealing the critical and especially key role of aquatic environments as a reservoir of resistance genes linking animal and human health [35]. This group of genes may originate from contamination of animals or human effluents due to the proximity to urban centers but may also supply genes to the food chain. As they encode resistance to antibiotics important to humans, this may thus jeopardize the effectiveness of chemotherapeutic treatment of bacterial infections or prophylaxis if ingested or integrated into the human microbiome.

Macrolides, tetracyclines, and oxazolidinones are medically important and used both in humans and animals [35]. Macrolides belong to category C (“Caution”) of the Antimicrobial Advice Ad Hoc Expert Group (AMEG) and veterinary critically important antimicrobials (VCIA) to the World Organization for Animal Health (OIE) and critically important AB—highest priority to World Health Organization (WHO). Tetracyclines belong to the category D (“Prudence”) AMEG and veterinary critically important antimicrobials (VCIA) to the OIE and highly important antimicrobials to WHO. Oxazolidinones belong to category A (“Avoid” prohibited) to AMEG and critically important AB—a high priority to the WHO. The OIE has no opinion on this class of antibiotics [35]. Resistance to disinfectants reveals a worrying effect of the increasing use of products on the adaptive and evolutionary response of microbial communities. This result agrees with our previous research on a dataset consisting of over 16,000 reference bacterial genomes that showed enrichment of the mobilome with genes encoding resistance to disinfectants [33]. These high levels of resistance genes and the fact that they tend to be encoded in the mobilome highlights, on the one hand, the selective pressure effect that bacteria and microbiomes are subjected to, but also the epidemic potential of these newly acquired traits.

### 4.2. Spreading Potential and Dissemination of ARs in Aquaculture Environments

We also tried to understand if there would be any link between AR within these microbial communities and their epidemic potential. A strong correlation between the total number of AR orthologues in the human gut microbiome (Mustard database [26]) and MGEs in the Sado Estuary was found. As the Mustard database gathers genes of bacteria from the human gut, this result highlights the horizontal transfer of AR genes from fecal contamination. Water contamination with antibiotic-resistant coliforms has already been reported in a large-scale international study [36] as well as the horizontal transfer of antibiotic-resistance genes of fecal origin in aquatic environments [37,38]. Their presence in these samples suggests that the mobilization of AR genes in MGEs is preserved in aquatic environments and links the gut microbiome to food production, and, eventually, back to the human gut through the food chain.

The diversity of genes encoding tetracycline resistance, although not positively correlated in the collection of the 17 samples from the Sado River, is clustered in two distinct groups (Figure 5 and Figure 6). When analyzing each of these groups of metagenomes separately, a strong correlation was found between the diversity of ARs and MGEs orthologues. The genomic diversification of genes involved in tetracycline resistance evidence a rapid evolution of these genes. The fact that the diversity of tetracycline-resistance encoding genes, (rather than the abundance) correlates with the diversity of MGEs is very worrying as it suggests that these may have been mobilized by different mobile genetic elements and new associations are being made. This trend is not as clear for genes encoding disinfectant resistance, the second most common resistance in this dataset. Although they have been identified in bacterial isolates along with mobile genetic elements [39], there is no clear correlation between the abundance and diversity of these two types of genetic elements in our metagenomes. This result contrasts with those for the tetracycline resistance ones which, in turn, may be a consequence of the overuse of these antibiotics in animal production.

## 5. Conclusions

Metagenomics can be used to address the genomic dynamics of AR transfer within and between microbial communities. This study allowed the identification of the resistance fingerprint of the Portuguese aquaculture farms located at three river estuaries, and the categories of AR genes worth monitoring, due to their higher frequency and critical importance to human health. The most diverse categories of antibiotic resistance belong to macrolide, tetracycline, and oxazolidinone classes.

The use of AR-specific databases gives insight into the origin of resistance in microbiomes and should be further explored. The results presented here, concerning the role of aquatic environments on the link between animal and environmental health with human health, are crucial for a complete understanding of the spread of antibiotic resistance genes.

## Figures and Tables

**Table 1:** Kruskal–Wallis and Dunn test statistics.

	Kruskal–Wallis (*p*-Value)	Dunn’s Test (Adjusted *p*-Value)	
Resfinder (Protein Diversity)	0.543	Aveiro–Lima	0.709
		Aveiro–Sado	0.905
		Lima–Sado	0.832
Resfinder (Genes)	0.034	Aveiro–Lima	0.677
		Aveiro–Sado	0.060
		Lima–Sado	0.240
Mustard (Protein diversity)	0.087	Aveiro–Lima	0.412
		Aveiro–Sado	0.082
		Lima–Sado	0.677
Mustard (Genes)	0.001	Aveiro–Lima	0.829
		Aveiro–Sado	0.004
		Lima–Sado	0.021

## Data Availability

The data presented in this study are openly available in MG-RAST at https://www.mg-rast.org/mgmain.html?mgpage=search&search=mgp95904 (accessed on 16 November 2022) and [21].

## Supplementary Materials

The following supporting information can be downloaded at: https://www.mdpi.com/article/10.3390/biology11111681/s1, Supplementary Figure S1: Distribution of the relative frequencies of orthologous antibiotic resistance genes and proteins; Table S1: Plasmids identified in metagenomes with PlasmidFinder for Sado River. File S1: Methods. The demultiplexing of the sequencing reads was performed with Illumina bcl2fastq (2.20), and adapters were trimmed with Skewer (version 0.2.2) [22]. The quality of the FASTQ files was analyzed with FastQC (version 0.11.5-cegat) [23]. The Illumina raw sequence data file pairs in the FASTQ format were assembled on the MG-RAST metagenomic analysis server [24].
