# Supplementary material for: Estuarine Aquacultures at the Crossroads of Animal Production and Antibacterial Resistance: A Metagenomic Approach to the Resistome
Source: Biology (Basel). 2022 Nov 21;11(11):1681. doi: 10.3390/biology11111681 (PMC9687122; doi:10.3390/biology11111681)
Supplement: Supplementary file 1 [file biology-11-01681-s001.zip › Figure S1.pdf]

Supplementary material

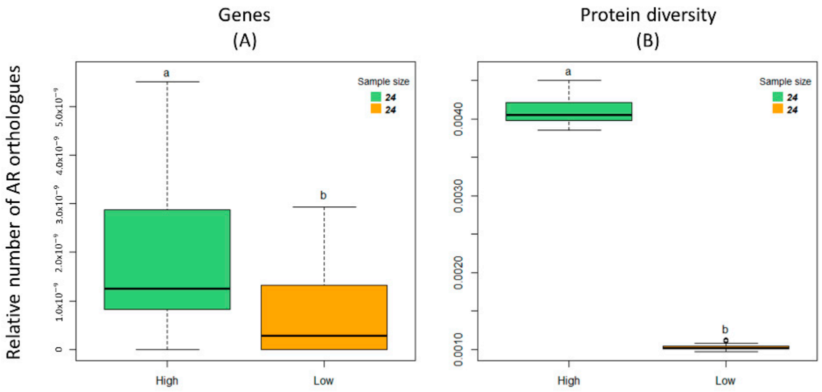

Figure S1. Orthologous antibiotic resistance genes divided according to their relative frequencies, obtained from ResFinder database (see Figure 3). (A) Genes, and (B) Protein diversity. Boxplots represent the sum of the highest relative frequencies (green) and the sum of the lowest relative frequencies (orange). In (A) the “High” group consists of aminoglycoside, beta-lactam, disinfectant, quinolone and tetracycline ResFinder categories, while in (B) the “High” group consists of disinfectant, macrolide, oxazolidinone and tetracycline ResFinder categories. The sample size of 24. The bottom and top of the boxplot are the first and third quartiles, the horizontal line is the median, and the vertical dashed lines are the 1.5 interquartile range. The black circles represent the outliers. A Wilcoxon rank sum test with continuity correction indicated that there are significant differences between the two groups, as indicated by the two different letters a and b (p-value < 0.05).
