# Supplementary material for: Estuarine Aquacultures at the Crossroads of Animal Production and Antibacterial Resistance: A Metagenomic Approach to the Resistome
Source: Biology (Basel). 2022 Nov 21;11(11):1681. doi: 10.3390/biology11111681 (PMC9687122; doi:10.3390/biology11111681)
Supplement: Supplementary file 1 [file biology-11-01681-s001.zip › Supplementary File S1-Methods.pdf]

Supplementary Material

Methods

At each site, samples were taken from the top 5 cm layer, stored in sterile polypropylene centrifuge tubes (10 ml), and immediately transported refrigerated to the laboratory where they were kept at -20ºC until analysis. The total DNA was extracted from the frozen sediment samples using the DNeasy PowerSoil Pro Kit (qiagen.com). DNA was sent to CeGaT (https://www.cegat.com/) in Germany in frozen isothermal boxes. Libraries for shotgun metagenome sequencing were prepared using an Illumina DNA (M) Tagmentation Library Prep kit from Illumina, followed by NovaSeq 6000, 2 × 100 bp sequencing. The demultiplexing of the sequencing reads was performed with Illumina bcl2fastq (2.20), and adapters were trimmed with Skewer (version 0.2.2) [22]. The quality of the FASTQ files was analyzed with FastQC (version 0.11.5-cegat) [23]. The Illumina raw sequence data file pairs in the FASTQ format were assembled on the MG-RAST metagenomic analysis server [24]. The pipeline options chosen were removing artificial replicate sequences, any host-specific Homo sapiens NCBI v36 specie sequence, and low-quality sequences. The lowest Phred scores to count as a high-quality base were set to 15 and trimmed at most, 5 low Phred score bases. All the resulting files can be assessed at https://www.mg-rast.org/mgmain.html?mgpage=search&search=mgp95904.

References

22. Jiang, H.; Lei, R.; Ding, S.-W.; Zhu, S. Skewer: A Fast and Accurate Adapter Trimmer for next-Generation Sequencing Paired-End Reads. *BMC Bioinformatics* **2014**, *15*, 182, doi:10.1186/1471-2105-15-182.

23. Babraham Bioinformatics - FastQC A Quality Control Tool for High Throughput Sequence Data Available online: https://www.bioinformatics.babraham.ac.uk/projects/fastqc/ (accessed on 14 November 2022).

24. Meyer, F.; Paarmann, D.; D'Souza, M.; Olson, R.; Glass, E.; Kubal, M.; Paczian, T.; Rodriguez, A.; Stevens, R.; Wilke, A.; et al. The Metagenomics RAST Server – a Public Resource for the Automatic Phylogenetic and Functional Analysis of Metagenomes. *BMC Bioinformatics* **2008**, *9*, 386, doi:10.1186/1471-2105-9-386.
