# Supplementary material for: Estuarine Aquacultures at the Crossroads of Animal Production and Antibacterial Resistance: A Metagenomic Approach to the Resistome
Source: Biology (Basel). 2022 Nov 21;11(11):1681. doi: 10.3390/biology11111681 (PMC9687122; doi:10.3390/biology11111681)
Supplement: Supplementary file 1 [file biology-11-01681-s001.zip › Table S1.pdf]

Supplementary material

Plasmid	Accession (GenBank)	Coverage	Identity	Note	HSP length	Template length
rep28	CP005948	10.79	10.68	*LBPp6g007(LBPp6)*	101	936
repUS39	AJ579365	19.86	18.98	*ORF14(pSCFS1)*	595	1017
Col(Ye4449)	FJ696405	12.37	12.37		24	194
IncQ1	M28829	18.22	18.22		145	796
repUS39	AJ579365	91.94	88.50	*ORF14(pSCFS1)*	938	1017
rep28	CP003162	14.62	14.41	*repA(pCIS4)*	241	930
repUS1	CP003585	19.4	19.0	*repE(DOp2)*	437	1500
rep7a	AM990993	13.47	13.33	*repC(pS0385p1)*	101	750
IncQ1	M28829	17.84	17.34		142	796

Table S1. Plasmids identified in our metagenomes with PlasmidFinder for Sado River. We have detected 6 different
